# Supplementary material for: PIKI-1, a class II phosphatidylinositol 3-kinase, functions in endocytic trafficking
Source: bioRxiv. 2025 May 23:2025.05.22.655458. Preprint. [Version 1] doi: 10.1101/2025.05.22.655458 (PMC12139958; doi:10.1101/2025.05.22.655458)
Supplement: 1 — S1 Fig. Supplemental RNAi and suppression data. (A) The proportion of viable progeny produced by nekl-2(fd81); nekl-3(gk894345) worms after injection by the indicated dsRNA. (B) The proportion of viable progeny produced by nekl-2(fd81); nekl-3(gk894345) piki-1(Q1507Stop) worms after injection by the indicated dsRNA. (C) The proportion of viable progeny produced by nekl-2(fd81); nekl-3(gk894345) worms for the indicated genotype. Statistical significance was determined using unpaired t-tests; *p≤0.05. Raw data available in S1 File. S2 Fig. Endogenous PIKI-1 expression. (A, B) Representative confocal images of day-1 adults expressing (A) PIKI-1::mScarlet and (B) PIKI-1::GFP. S3 Fig. Effects of reduction of PIKI-1 function on intracellular trafficking compartments and cargo. (A,B,D,E,G,H,K,L,O,P) Representative confocal microscopy images of day-1 adults to assess the effects of piki-1(Q1507Stop) mutants relative to wild-type worms with respect to (A,B) GFP::CHC-1 (n=93), (D,E) mScarlet::CLIC-1 (n=79), (G,H) Phyp7::LRP-1::GFP (n=31), (K,L) Phyp7::GFP::RAB-11, and (O,P) Phyp7::mNeonGreen::LGG-1. Red arrows in (P) indicate instances of tubulation. The seam cell is labeled in all images. (C, F, I, M, Q) The mean intensity for each marker tested in (A,B) GFP::CHC-1, (D,E) mScarlet::CLIC-1, Phyp7::LRP-1::GFP, (K,L) Phyp7::GFP::RAB-11, and (O,P) Phyp7::mNeonGreen::LGG-1. (J) The mean intensity for Phyp7::TGN-38::GFP. (N) The size of vesicles was graphed onto a dot plot for the Phyp7::GFP::RAB-11 strains. (R) The percentage of worms that had either no tubulations or tubulations was recorded for Phyp7::mNeonGreen::LGG-1. (C, F, I, J, M, N, Q) Dot plots show the mean and 95% CI. Statistical significance was determined by unpaired t-tests; ****p ≤ 0.0001, **p ≤ 0.01, *p ≤ 0.05; ns, not significant. (R) Statistical significance was determined by Fisher’s exact test; ****p ≤ 0.0001. Raw data are available in S1 File. S4 Fig. Effects of piki-1(ok2346) deletion allele on PI(3)P and PI [file NIHPP2025.05.22.655458V1-supplement-1.pdf]

bioRxiv preprint doi: https://doi.org/10.1101/2025.05.22.655458; this version posted May 23, 2025. The copyright holder for this preprint (which was not certified by peer review) is the author/funder, who has granted bioRxiv a license to display the preprint in perpetuity. It is made available under aCC-BY 4.0 International license.

Supporting Information

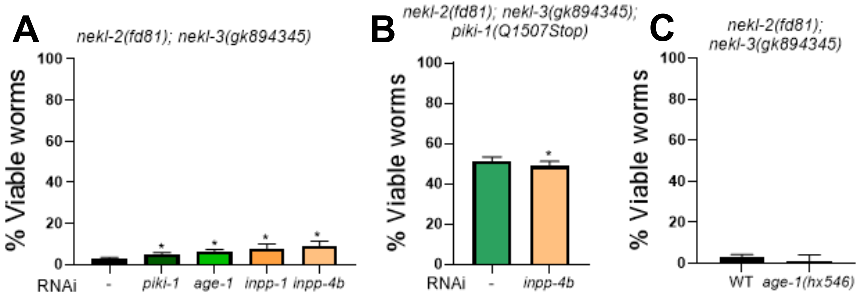

S1 Fig. Supplemental RNAi and suppression data.

(A) The proportion of viable progeny produced by *nekl-2(fd81); nekl-3(gk894345)* worms after injection by the indicated dsRNA. (B) The proportion of viable progeny produced by *nekl-2(fd81); nekl-3(gk894345) piki-1(Q1507Stop)* worms after injection by the indicated dsRNA. (C) The proportion of viable progeny produced by *nekl-2(fd81); nekl-3(gk894345)* worms for the indicated genotype. Statistical significance was determined using unpaired t-tests; *p≤0.05. Raw data available in S1 File.

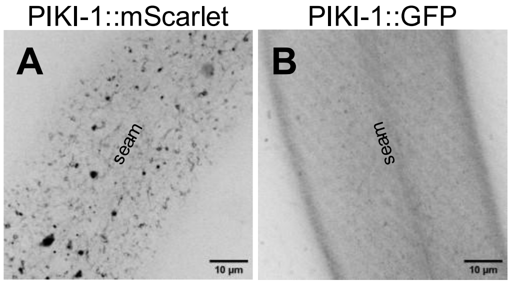

S2 Fig. Endogenous PIKI-1 expression.

(A, B) Representative confocal images of day-1 adults expressing (A) PIKI-1::mScarlet and (B) PIKI-1::GFP.

bioRxiv preprint doi: https://doi.org/10.1101/2025.05.22.655458; this version posted May 23, 2025. The copyright holder for this preprint (which was not certified by peer review) is the author/funder, who has granted bioRxiv a license to display the preprint in perpetuity. It is made available under aCC-BY 4.0 International license.

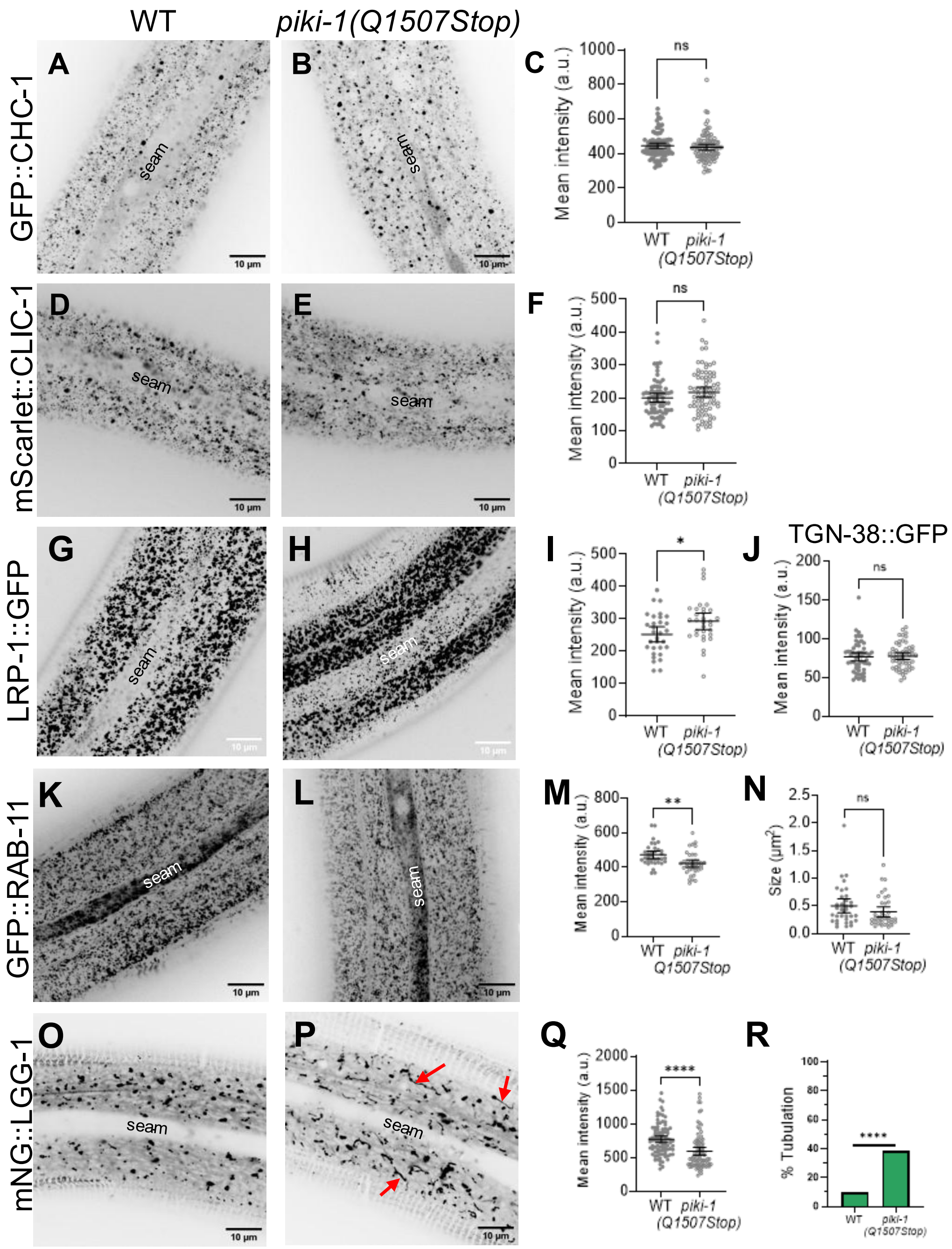

S3 Fig. Effects of reduction of PIKI-1 function on intracellular trafficking compartments and cargo.

(A,B,D,E,G,H,K,L,O,P) Representative confocal microscopy images of day-1 adults to assess the effects of *piki-1(Q1507Stop)* mutants relative to wild-type worms with respect to (A,B) GFP::CHC-1 (n=93), (D,E) mScarlet::CLIC-1 (n=79), (G,H) P_{hyp7}::LRP-1::GFP (n=31), (K,L) P_{hyp7}::GFP::RAB-11, and (O,P) P_{hyp7}::mNeonGreen::LGG-1. Red arrows in (P) indicate instances of tubulation. The seam cell is labeled in all images. (C, F, I, M, Q) The mean intensity for each

bioRxiv preprint doi: https://doi.org/10.1101/2025.05.22.655458; this version posted May 23, 2025. The copyright holder for this preprint (which was not certified by peer review) is the author/funder, who has granted bioRxiv a license to display the preprint in perpetuity. It is made available under aCC-BY 4.0 International license.

marker tested in (A,B) GFP::CHC-1, (D,E) mScarlet::CLIC-1, P_{hyp7}::LRP-1::GFP, (K,L) P_{hyp7}::GFP::RAB-11, and (O,P) P_{hyp7}::mNeonGreen::LGG-1. (J) The mean intensity for P_{hyp7}::TGN-38::GFP. (N) The size of vesicles was graphed onto a dot plot for the P_{hyp7}::GFP::RAB-11 strains. (R) The percentage of worms that had either no tubulations or tubulations was recorded for P_{hyp7}::mNeonGreen::LGG-1. (C, F, I, J, M, N, Q) Dot plots show the mean and 95% CI. Statistical significance was determined by unpaired *t*-tests; ****$p \leq 0.0001$, **$p \leq 0.01$, *$p \leq 0.05$; ns, not significant. (R) Statistical significance was determined by Fisher's exact test; ****$p \leq 0.0001$. Raw data are available in S1 File.

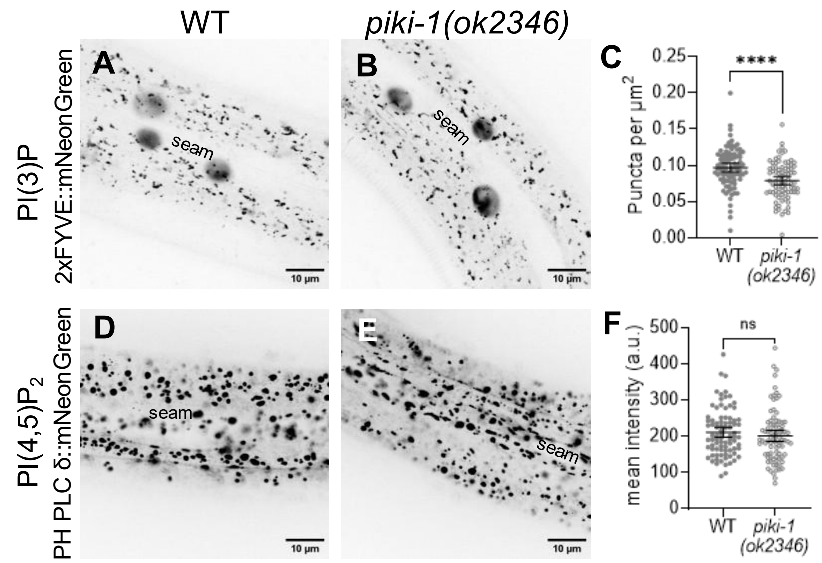

S4 Fig. Effects of *piki-1(ok2346)* deletion allele on PI(3)P and PI(4,5)P_2.

(A,B,D,E) Representative confocal images of day-1 adults show the effects in *piki-1(ok2346)* mutants relative to wild-type worms with respect to (A, B) the PI(3)P lipid sensor P_{hyp7}::2xFYVE::mNeonGreen and (D,E) the PI(4,5)P_2 lipid sensor P_{hyp7}::PH PLC δ::mNeonGreen. (C) Puncta per unit area for worms expressing P_{hyp7}::2xFYVE::mNeonGreen. (F) Mean intensity for worms expressing P_{hyp7}::PH PLC δ::mNeonGreen. Dot plots show the mean and 95% CI. Statistical significance was determined by unpaired *t*-tests; ****$p \leq 0.0001$; ns, not significant. Raw data are available in S1 File.

bioRxiv preprint doi: https://doi.org/10.1101/2025.05.22.655458; this version posted May 23, 2025. The copyright holder for this preprint (which was not certified by peer review) is the author/funder, who has granted bioRxiv a license to display the preprint in perpetuity. It is made available under aCC-BY 4.0 International license.

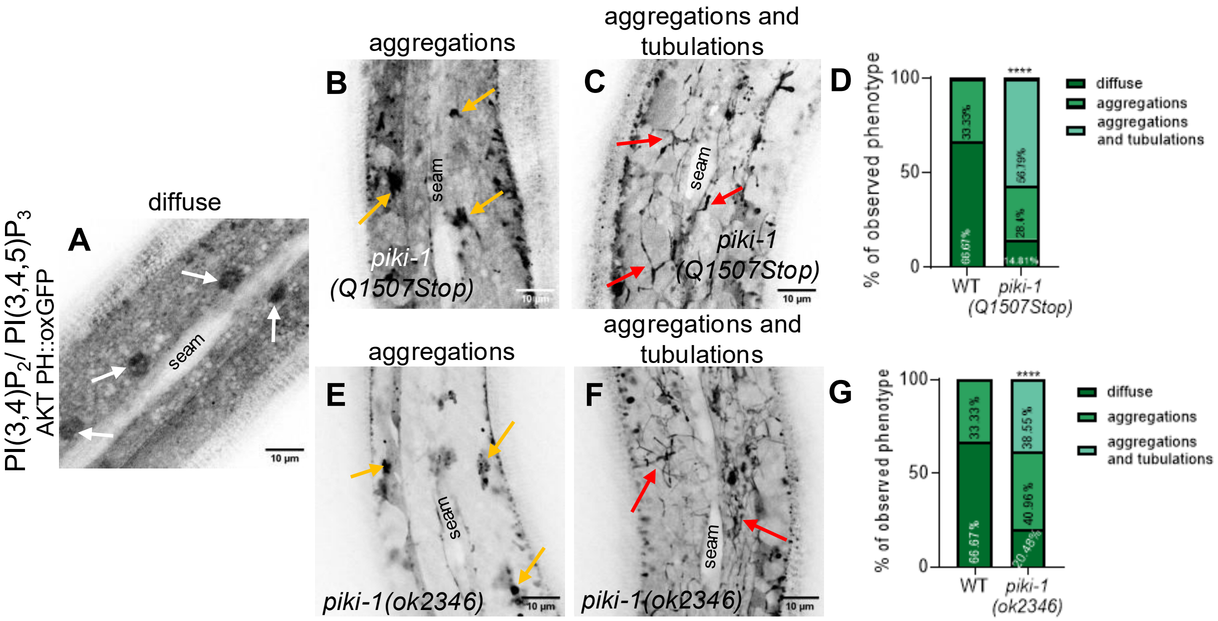

S5 Fig. Effects of reduction of PIKI-1 function on the multi-specific lipid sensor AKT-PH.
(A–C,E,F) Representative confocal images of day-1 adults that expressed the $PI(3,4)P_2/PI(3,4,5)P_3$ lipid sensor P_{hyp7}::AKT::oxGFP in the (A) wild-type, (B,C) *piki-1(Q1507Stop)* (n=81), or (E,F) *piki-1(ok2346)* (n = 83) background. White arrows (A) indicate nuclei. Gold arrows (B, E) indicate aggregations. Red arrows (C, F) indicate tubulations. (D, G) Worms expressing P_{hyp7}::AKT-PH::oxGFP were scored for the presence of diffuse labeling, of aggregations, and of aggregations and tubulations within the epidermis. Statistical significance of the differences in phenotype distribution across backgroundswas determined by Fisher's exact test; ****p < 0.0001. Raw data are available in S1 File.

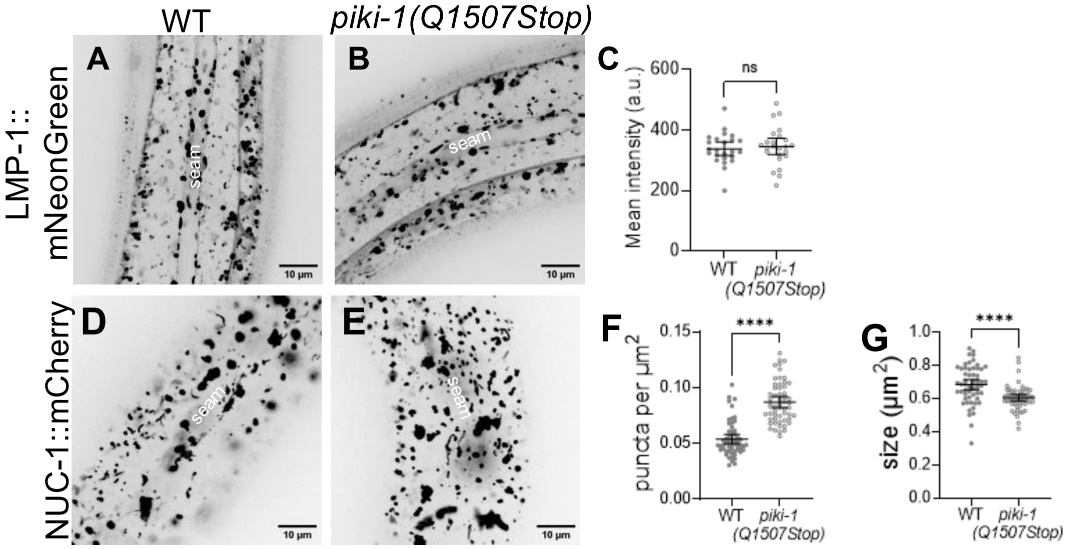

bioRxiv preprint doi: https://doi.org/10.1101/2025.05.22.655458; this version posted May 23, 2025. The copyright holder for this preprint (which was not certified by peer review) is the author/funder, who has granted bioRxiv a license to display the preprint in perpetuity. It is made available under aCC-BY 4.0 International license.

S6 Fig. Effects of reduction of PIKI-1 function on lysosomes.

(A,B,D,E) Representative confocal images of day-1 adults expressing (A,B) P_{hyp7}::LMP-1::mNeonGreen (n=24) or (D,E) NUC-1::mCherry (n=56) in the (A,D) wild-type and (B,E) *piki-1(Q1507Stop)* backgrounds. (C) Mean intensity was plotted for P_{hyp7}::LMP-1::mNeonGreen. (F,G) The (F) number of puncta per unit area and (G) size were plotted for NUC-1::mCherry. Dot plots show the mean and 95% CI. Statistical significance was determined by unpaired *t*-tests; ****p ≤ 0.0001; ns, not significant.

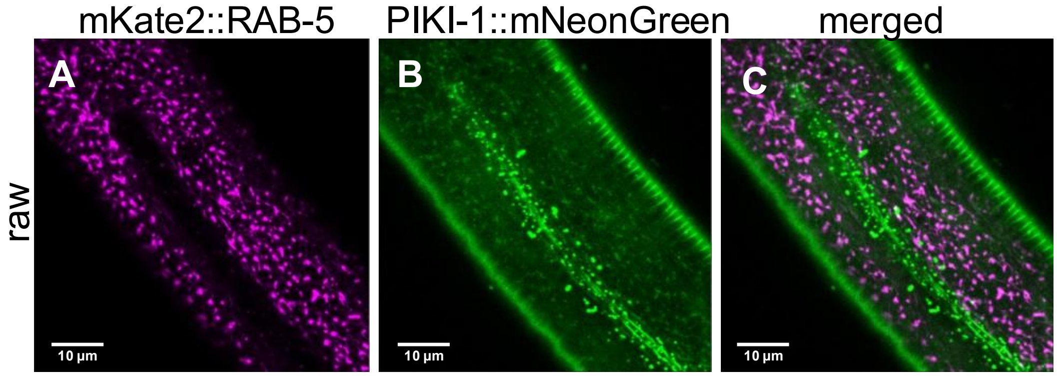

S7 Fig. Marker expression in worms homozygous for P_{hyp7}::PIKI-1::mNeonGreen and $P_{dyp\text{-}7}$::mKate2::RAB-5.

(A–C) Representative raw images of a day-1 adult homozygous for both P_{hyp7}::PIKI-1::mNeonGreen and $P_{dpy\text{-}7}$::mKate2::RAB-5. Both (A,B) single-channel and (C) merged images are shown.

S1 Movie. $P_{nekl\text{-}3}$::2xTAPP-1::mNeonGreen in the epidermis of a wild-type worm.

z-Stack movie showing the expression of the PI(3,4)P_2 sensor $P_{nekl\text{-}3}$::2xTAPP-1::mNeonGreen from the apical to the basal plane in the epidermis of a wild-type worm.

S2 Movie. $P_{nekl\text{-}3}$::2xTAPP-1::mNeonGreen in the epidermis of a *piki-1(Q1507Stop)* worm.

z-Stack movie showing the expression of the PI(3,4)P_2 sensor $P_{nekl\text{-}3}$::2xTAPP-1::mNeonGreen from the apical to the basal plane in the epidermis of a *piki-1(Q1507Stop)* mutant.
